# Development of Two Novel One-Step and Green Microwell Spectrophotometric Methods for High-Throughput Determination of Ceritinib, a Potent Drug for Treatment of Anaplastic Lymphoma Kinase-Positive Non-Small-Cell Lung Cancer

**DOI:** 10.3390/medicina59101813

**Published:** 2023-10-12

**Authors:** Reem M. Abuhejail, Nourah Z. Alzoman, Ibrahim A. Darwish

**Affiliations:** Department of Pharmaceutical Chemistry, College of Pharmacy, King Saud University, P.O. Box 2457, Riyadh 11451, Saudi Arabia

**Keywords:** ceritinib, microwell analysis, spectrophotometry, green analytical approach, high-throughput analysis

## Abstract

*Background and Objectives:* Ceritinib (CER) is a potent drug of the third-generation tyrosine kinase inhibitor class. CER has been approved for the treatment of patients with non-small-cell lung cancer (NSCLC) harboring the anaplastic lymphoma kinase (ALK) mutation gene. In the literature, there is no green and high-throughput analytical method for the quantitation of CER in its dosage form (Zykadia^®^ capsules). This study describes, for the first time, the development and validation of two novel one-step and green microwell spectrophotometric methods (MW-SPMs) for the high-throughput quantitation of CER in Zykadia^®^ capsules. *Materials and Methods:* These two methods were based on an *in microwell* formation of colored derivatives upon the reaction of CER with two different benzoquinone reagents via two different mechanisms. These reagents were *ortho*-benzoquinone (OBQ) and 2,3-dichloro-5,6-dicyano-1,4-benzoquinone (DDQ), and their reactions proceeded via condensation and charge transfer reactions, respectively. The reactions were carried out in 96-well transparent plates, and the absorbances of the colored reaction products were measured with an absorbance microplate reader at 540 and 460 nm for reactions with OBQ and DDQ, respectively. The optimum conditions of reactions were established, their molar ratios were determined, and reaction mechanisms were postulated. Under the refined optimum reaction conditions, procedures of MW-SPMs were established and validated according to the guidelines of the International Council on Harmonization. *Results:* The limits of quantitation were 6.5 and 10.2 µg/well for methods involving reactions with OBQ and DDQ, respectively. Both methods were applied with great reliability to the determination of CER content in Zykadia^®^ capsules and their drug uniformity. Greenness of the MW-SPMs was evaluated using three different metric tools, and the results proved that the two methods fulfil the requirements of green analytical approaches. In addition, the simultaneous handling of a large number of samples with microvolumes in the proposed methods gave them the advantage of a high-throughput analysis. *Conclusions*: The two methods are valuable tools for rapid routine application in pharmaceutical quality control units for the quantitation of CER.

## 1. Background and Objectives

Non-small-cell lung cancer (NSCLC) is the most common type of lung cancer, accounting for approximately 85% of all lung cancer cases. It is a malignant condition that primarily affects the cells lining the airways of the lungs [1]. For advanced metastatic and/or localized NSCLC, chemotherapy is conducted with first-generation tyrosine kinase inhibitors (TKIs) [2,3,4]; however, these drugs are not effective in NSCLC cases harboring the gene resulting from the fusion of echinoderm microtubule-associated protein-like 4 (EML4) with anaplastic lymphoma kinase (ALK) genes [4,5]. Building on this discovery, pharmaceutical companies have embarked on research programs to discover and develop effective ALK inhibitors.

Ceritinib (CER) was identified as a potent ALK inhibitor during drug discovery efforts led by Novartis Pharmaceutical Corporation (Basel, Switzerland). The chemical name of CER is 5-chloro-N^2^-{5-methyl-4-(piperidin-4-yl)-2-[(propan-2-yl)oxy]phenyl}-N^4^-[2-(propane-2-sufonyl)phenyl]pyrimidine-2,4-diamine [6]. CER was designed to overcome resistance to the earlier ALK inhibitor, crizotinib. CER has been approved by the U.S. Food and Drug Administration (FDA) as a breakthrough treatment option for patients with ALK-positive NSCLC. CER has been marketed under the trade name of Zykadia^®^ capsules [7]. Since its approval, CER has shown remarkable efficacy and safety, offering renewed hope for patients with this specific subtype of lung cancer. CER has the capability to inhibit multiple receptor tyrosine kinases, including ALK, ROS1, and insulin-like growth factor 1 receptor (IGF-1R). By specifically targeting and blocking the activity of these kinases, CER effectively suppresses the growth and survival of cancer cells harboring ALK gene rearrangements. This targeted approach makes CER a highly specific and remarkably impressive potent therapy for ALK-positive NSCLC [8].

While CER has demonstrated substantial clinical benefits, it shows some adverse events, including gastrointestinal disturbances, hepatotoxicity, fatigue, and edema. These side effects can be manageable with dose modifications; however, CER has been associated with QT interval prolongation (an extended interval between the heart contracting and relaxing), necessitating careful monitoring and appropriate dose management [8]. For these reasons, thorough control of CER content in its dosage form (Zykadia^®^ capsules) is very important in achieving an effective and safe therapy with CER. To achieve this goal, an efficient and reliable analytical tool is required for quantitation of CER in its capsules.

Few methods are available for the characterization and/or quantitation of CER in bulk drug or dosage form. These methods are liquid chromatography coupled with an ultraviolet detector (HPLC-UV) [9,10] or a tandem mass spectrometric detector (LC-MS/MS) [11]. HPLC-UV methods were developed for the stability testing of CER; however, LC-MS/MS methods were developed for the quantitation of genotoxic impurities in CER bulk form [12]. Despite liquid chromatographic methods offering versatility in certain cases, the technique has many disadvantages and limitations when applied in pharmaceutical quality control laboratories for drug formulation characterization. These disadvantages include (1) complexity, as the technique requires a high level of expertise to operate the instrument, optimize the method, and interpret the results accurately; (2) high cost for setting up and maintaining the system, as well as consuming; (3) challenges posed in high-throughput situations requiring rapid processing of many samples, such as dosage form uniformity testing; and (4) solvent disposal challenges because of using organic solvents, which can be hazardous and environmentally unfriendly. To overcome these disadvantages, the development of better alternative methodology is essential. 

Microwell spectrophotometric methods (MW-SPMs) assisted with absorbance microplate readers have a great importance in the field of pharmaceutical analysis because the methodology provides extraordinary advantages [12,13,14,15]. The main benefit of MW-SPMs is the use of smaller sample volumes than the conventional spectrophotometric technique, which uses volumetric flasks and cuvettes. This approach reduces the amount of waste generated during the analytical process and lowers the cost of reagents. Furthermore, it can reduce the amounts of hazardous solvents and/or chemicals, thereby enhancing the eco-friendliness and greenness of the process. The technique is versatile and can be used for a wide range of applications in pharmaceutical industry activities. It can be easily automated using robotic systems, which can enhance efficiency, reduce errors, and save time and labor in the laboratory. The application of these methods in pharmaceutical analysis enables the rapid analysis of numerous samples, ultimately achieving uniformity in pharmaceutical formulations and other activities within the pharmaceutical industry. These advantages have made the technique increasingly popular in the pharmaceutical industry and beyond. 

This study describes, for the first time, the development of two different MW-SPMs for quantitation of CER in Zykadia^®^ capsules. Both methods involve the *in microwell* formation of colored benzoquinone derivatives of CER upon its reactions with *ortho*-benzoquinone (OBQ) and 2,3-dichloro-5,6-dicyano-1,4-benzoquinone (DDQ) reagents. The reaction with OBQ is a condensation reaction of CER via its piperidinyl secondary amino group. The reaction with DDQ is a charge transfer (CT) reaction with CER as an electron donor and DDQ as a π-electron acceptor. Both reactions are conducted in transparent 96-well plates, and the absorbances of reaction’s solutions are measured with an absorbance microplate reader at 540 and 460 nm for reactions with OBQ and DDQ, respectively. Both MW-SPMs methods meet the requirements of high-throughput and green analytical approaches used in the pharmaceutical industry.

## 2. Materials and Methods

### 2.1. Apparatus

A double-beam ultraviolet-visible spectrophotometer (model V-530; JASCO Co., Ltd., Kyoto, Japan) was used for scanning the absorption spectra of CER and its reaction mixtures. An absorbance microplate reader (model ELx808; Bio-Tek Instruments Inc., Winooski, VE, USA) was controlled with KC junior software (version 2.0) provided with the reader. 

### 2.2. Chemicals and Materials

CER was bought from LC Laboratories (Woburn, MA, USA); its purity was ≥99%. Zykadia^®^ capsules (Batch no. 0078-0694-84, Novartis Pharmaceutical Corporation, Basel, Switzerland) were kindly donated by the Saudi FDA (Riyadh, Saudi Arabia) and were labeled to contain 150 mg of CER per capsule. OBQ was prepared via the oxidation of catechol with silver oxide according to the procedures previously described by our laboratory [16]. The OBQ solution was 0.5% (*w*/*v*, in methanol). DDQ was purchased from Sigma-Aldrich Chemicals Co. (St. Louis, MO, USA). The DDQ solution was 1% (*w*/*v*, in methanol). Corning^®^ 96-well transparent polystyrene assay plates with flat bottoms were purchased from Merck & Co., Inc. (Rahway, New Jersey, NJ, USA). Adjustable single- and 8-channel pipettes (Finnpipette™) were obtained from Thermo Fisher Scientific Inc. (Waltham, MA, USA). Reagent reservoirs (BRAND^®^ PP) with cover lids for dispensing the solutions with the 8-channel pipettes were purchased from Merck KGaA (Darmstadt, Germany). All solvents were of spectroscopic grade (Merck, Darmstadt, Germany). All other chemicals used throughout the work were of analytical grade. 

### 2.3. Preparation of Solutions

#### 2.3.1. Standard CER Solution

An accurately weighed quantity (20 mg) of CER powder was dissolved in 10 mL of methanol. This stock solution (2 mg/mL) was diluted with methanol to yield working solutions in concentration ranges of 5–150 and 10–200 µg/mL for analysis of reaction with OBQ and DDQ, respectively.

#### 2.3.2. Capsule Sample Solution

The contents of 10 Zykadia^®^ capsules were collected, and an accurate weight of the contents equivalent to 50 mg of CER was transferred to a 25 mL calibrated flask. An amount of 20 mL of methanol was added, and the contents were shaken well to ensure the complete dissolution of CER. The flask’s content was filtered via passing through a 0.4 μm membrane filter. The filtrate was diluted with methanol for analysis of MW-SPMs via reactions with OBQ and DDQ. 

### 2.4. Recommended Procedures of MW-SPMs 

Aliquots (100 μL) of the CER standard or capsule sample solutions of varying concentrations were dispensed in each well of transparent 96-microwell plates. The CER concentration ranges were 5–150 and 10–200 µg/mL for analysis of reaction with OBQ and DDQ, respectively. An amount of 100 μL of OBQ or DDQ was dispensed into each well, and the reactions were allowed to proceed for 5 and 10 min for reactions with OBQ and DDQ, respectively. Absorbance of the reaction solution in each well was measured using an absorbance microplate reader at 540 and 460 nm for reactions with OBQ and DDQ, respectively. Wells containing 200 μL of methanol were used as blanks, and the measured absorbances of the blank wells were subtracted from those of the sample wells.

### 2.5. Determination of Reaction Molar Ratio 

Job’s method [17] of continuous variation was employed for determination of the molar ratio of CER reactions with both OBQ and DDQ. Master equimolar (2 × 10^−3^ mol/L) solutions of CER, OBD, and DDQ were prepared. Series of CER and OBQ or DDQ were used to make up different complementary reaction proportions of CER:reagent (0:00, 25:175, 50:150, 100:100, 125:75, 150:50, 175:25, and 200:0). The reactions were proceeded as described under the recommended procedures for MW-SPMs, and the absorbances were measured. The measured absorbances were plotted as a function [CER]/[Reagent], and molar ratios of CER:reagent were determined from the generated plots.

### 2.6. Content Uniformity Testing for Zykadia^®^ Capsules

The content uniformity testing was carried out according to the procedures described by the United States Pharmacopeia (USP) [18] for content uniformity testing of Zykadia^®^ capsules. Briefly, 10 capsules were analyzed using the two proposed MW-SPMs, and the CER content of each individual capsule and the mean content of the 10 capsules were measured. The acceptance value was calculated and compared with that of the maximum allowable value for content uniformity testing of 10 units of solid dosage form [18].

## 3. Results and Discussion

### 3.1. Strategy for Selection of Reagents and Reactions

**Figure 1:**
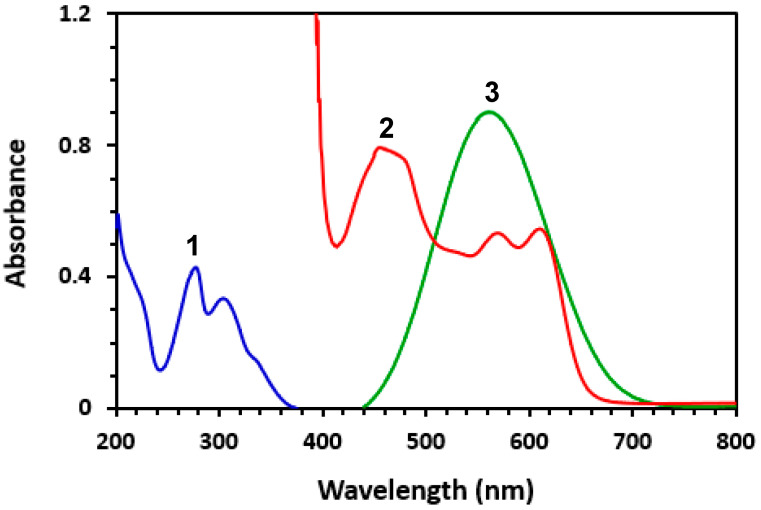
The absorption spectra of CER (1), reaction mixture of CER with DDQ (2), and reaction mixture of CER with OBQ (3). The concentration of the CER solution alone (without reaction) was 10 µg/mL. The concentrations of CER in its reactions with DDQ (1%, *w*/*v*) and OBQ (0.5%, *w*/*v*) were 30 and 50 µg/mL, respectively. All solutions were in methanol.

The UV absorption spectrum of CER (Figure 1) showed a maximum absorption peak (λ_max_) at 280 nm. Because of this blue-shifted λ_max_ of CER, its quantitation in Zykadia^®^ capsules via measuring UV absorption may pose potential interferences from the co-extracted inactive excipients used in the formulation of capsules. Therefore, derivatization of CER to more red-shifted colored derivatives was necessary. 

The chemical structure of CER contains two secondary amino groups and one piperidinyl amino group. These amino groups could be reacted with many color-producing reagents [19,20]. Among these reagents, *ortho*-benzoquinone (OBQ) has 2 *ortho*-carbonyl groups that can exhibit a condensation reaction with secondary amino groups of CER. The use of OBQ offers versatile condensation reactions with a wide range of amines under very mild conditions. Its versatility and mild reaction conditions allow its successful use as a color-producing reagent for the analysis of different amines [16,21]. Additionally, the condensation reaction between OBQ and amines typically proceeds with high yields, ensuring efficient conversion of amine analytes into the desired colored reaction products. Because of these advantages, OBQ was selected in the present study for reaction with CER.

CER also contains additional multiple-electron-donating sites on its chemical structure. These sites include amino group nitrogen atoms, ether-oxygen atoms, and sulphone oxygen atoms. These electron-rich atoms are anticipated to contribute to the formation of colored charge transfer (CT) complexes with different electron acceptors [22]. Among these acceptors, DDQ is the most widely used substituted benzoquinone electron acceptor with a high electron affinity [23], and it commonly mediates easy and instantaneous CT reactions with a wide range of electron donors [24]. Therefore, DDQ was selected in the present study for investigating its CT reaction as a basis for spectrophotometric quantitation of CER. 

### 3.2. Methodology Selection and Design

The proposed MW-SPMs for CER were designed to employ a 96-microwell analysis assisted with an absorbance microplate reader. This methodology has emerged as a valuable tool in the pharmaceutical analysis field due to its ability to meet the requirements of green chemistry approaches [25,26,27] and high-throughput analysis [28,29]. The methodology provides a combination of many advantages, such as miniaturization, automation, and parallelization, achieving significant advancements in both environmental sustainability and efficient analysis of large sample sets [30,31,32]. Microwell analyses contribute to green chemistry in several ways, including (1) reduced sample and reagent consumption, which leads to less waste generation and lower environmental impact; (2) efficient energy utilization because the miniaturized simultaneous analysis nature of microwell methods allows for faster and more efficient analysis; and (3) microwell assays amenable to automation, allowing for high precision and reproducibility of analytical results via minimizing of human error. Regarding high-throughput screening, microwell methods offer several advantages, including (1) parallelization of measurements, allowing for simultaneous analysis of multiple samples, which greatly increases throughput and enables the analysis of a large number of samples in a single experiment; (2) miniaturized format of microwell methods using small sample and reagent volumes, enabling the screening of a vast number of samples with limited resources; and (3) ease of data handling and analysis because the large datasets generated via microwell analysis can be efficiently handled and analyzed using automated data analysis software. Because of these advantages, the present study undertook the development of MW-SPMs for quantitation of CER in its capsules. The proposed MW-SPMs for CER were designed to include carrying out the reactions of CER with OBQ and DDQ in transparent 96-well plates, and the absorbances were measured simultaneously with an absorbance microplate reader. 

### 3.3. Absorption Spectra and Nature of Reactions 

Upon mixing CER with OBQ and DDQ, the reaction mixtures turned violet and red, respectively. The absorption spectra of reaction mixtures were recorded against reagent blanks. The spectra showed maximum absorption peaks (λ_max_) at 540 and 460 nm for reaction mixtures of OBQ and DDQ, respectively (Figure 1). Obviously, the λ_max_ of both CER-OBQ and CER-DDQ derivatives were significantly red-shifted from that of the underivatized CER. This shift enabled the measurements in the visible region and eliminated the potential interference from inactive capsule ingredients. It was also found that the absorption intensities of these bands increased as the concentrations of CER increased. The appearance of these new bands and dependence of their intensities on CER concentrations revealed the occurrence of reactions between CER and OBQ or DDQ. The shapes and patterns of these new absorption bands were identical to those found in the literature for the condensation reaction products of amines with OBQ [16,21] and the CT reactions of electron donors with DDQ [32,33]. This observation revealed that the reactions of CER with OBQ and DDQ were condensation and CT reactions, respectively. For further confirmation of the nature of these reactions, the reaction mixtures were turned acidic by adding dilute mineral acid (HCl) to both reactions. Upon acidification of the reaction mixtures, the violet color of the reaction mixture of OBQ was not affected; however, the red color of the reaction mixture with DDQ disappeared, indicating its reversibility in an acidic medium. These results confirmed the nature of reactions with OBQ and DDQ as condensation and CT reactions, respectively.

### 3.4. Optimum Reaction Conditions

**Figure 2:**
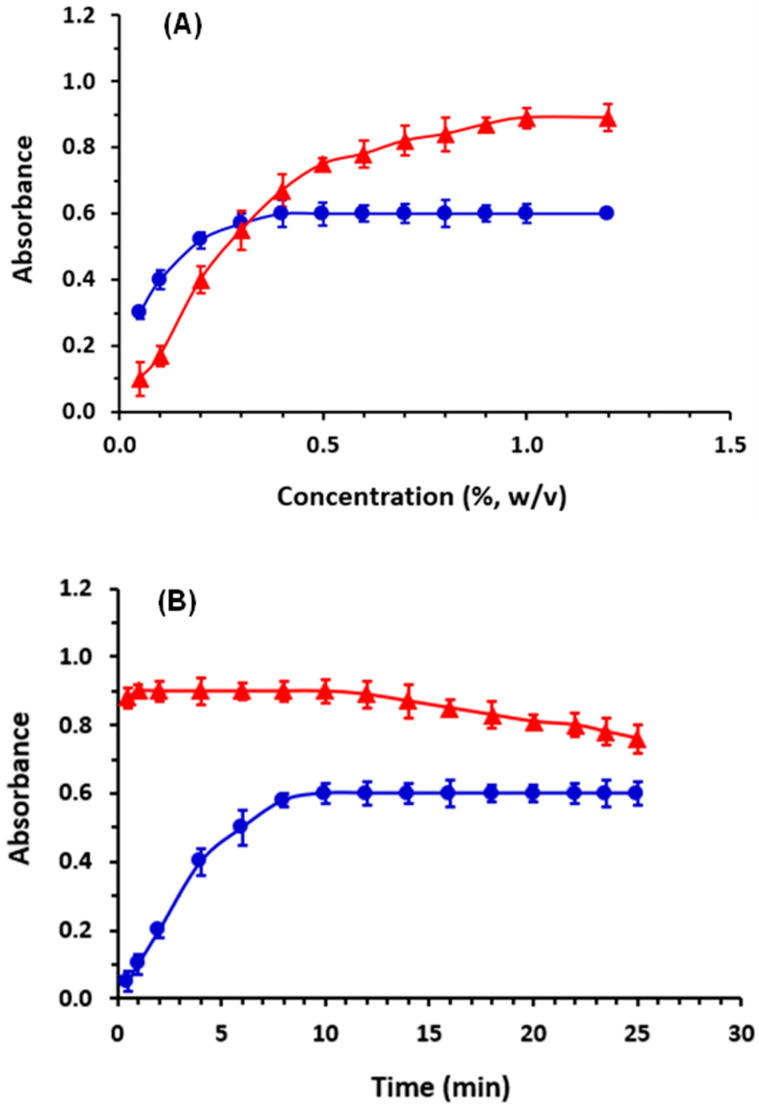
The effect of reagent concentration (**A**) and reaction time (**B**) on the absorbances of reactions of CER with OBQ (●) and DDQ (▲). The concentrations of CER in its reactions with OBQ and DDQ were 60 and 100 50 µg/well, respectively. The reactions were carried out in methanol. For panel (**A**), the time point used for measuring the absorbance was 10 min. For panel (**B**), the concentrations of OBQ and DDQ were 0.5 and 1 (%, *w*/*v*), respectively.

The effect of OBQ and DDQ concentration was studied via conducting the reactions using varying concentrations of each reagent (0.05–1.2%, *w*/*v*). The results revealed that the absorbances of reaction solutions increased as the reagent concentration increased (Figure 2A). The absorbances reached their maximum values when OBQ and DDQ concentrations were 0.5 and 1% (*w*/*v*), respectively. Beyond these concentrations, the absorbances did not increase. Therefore, subsequent experiments were carried out using OBQ and DDQ at concentrations of 0.5 and 1% (*w*/*v*), respectively.

**Figure 3:**
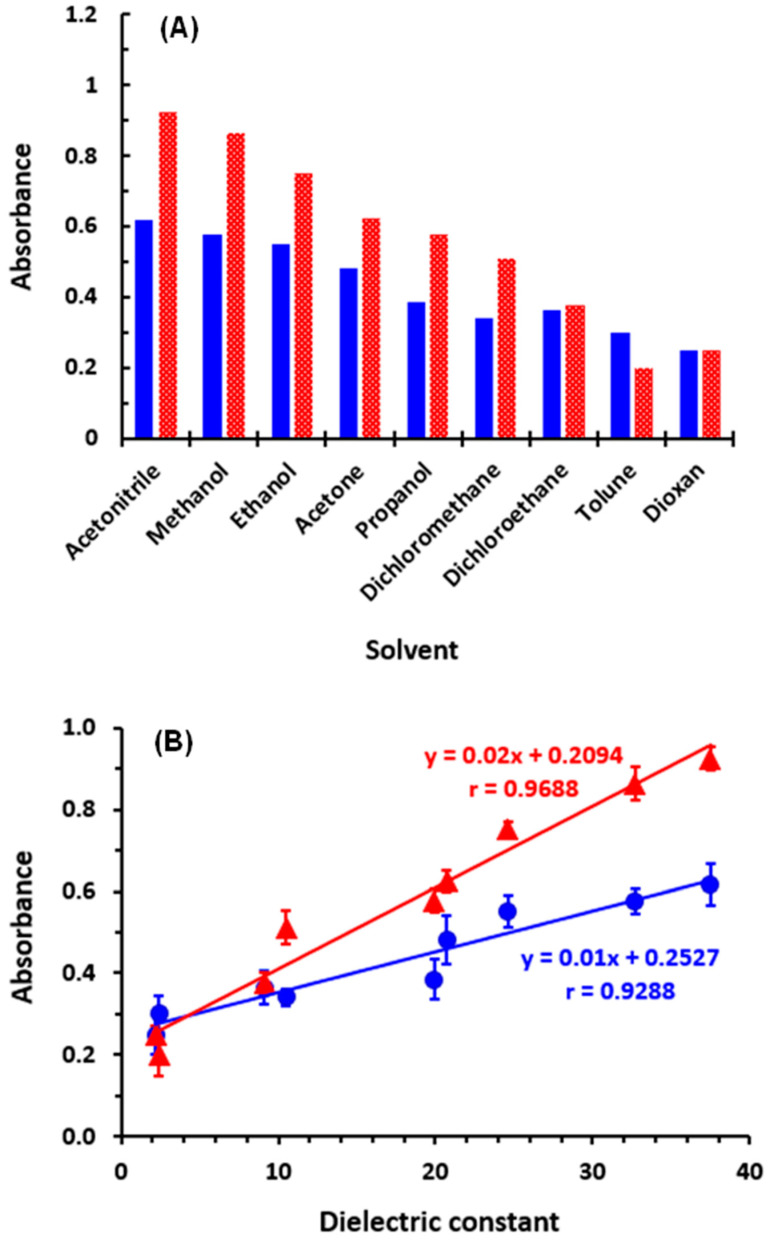
The effect of the type of solvent on the reactions of CER with OBQ and DDQ. Panel (**A**): the absorbances of the reactions of CER with OBQ (solid blue bars) and DDQ (dotted red bars) are plotted as a function of solvent name. Panel (**B**): the absorbances the of the reaction mixtures of CER with OBQ (●) and DDQ (▲) are plotted as a function of the dielectric constant of the solvents. The dielectric constants of the solvents were 37.5 (acetonitrile), 32.7 (methanol), 24.6 (ethanol), 20.7 (acetone), 19.9 (propanol), 10.5 (dichloromethane), 9.1 (dichloroethane), 2.4 (toluene), and 2.2 (dioxane).

The reaction between CER and DDQ was found to be instantaneous at 25 ± 2 °C, and the resulting absorbances of color solutions were stable for 10 min, after which the absorbances slightly decreased (Figure 3B). Although the reaction was instantaneous, measurements in the subsequent experiments were carried out after 5 min for obtaining better reading precision than measurements conducted directly after the mixing of CER with DDQ. The reaction of CER with OBQ was not instantaneous like DDQ, as obvious from the results presented in Figure 2B. The absorbances of the reaction solutions increased parallelly with reaction time and reached their maximum values after 10 min. Longer time did not lead to any further increase in the absorbances. Accordingly, measurements of the subsequent experiments were carried after 10 min from the starting of the reaction.

To determine the most appropriate solvent for carrying out reactions of CER with OBQ and DDQ, the reactions were conducted in different solvents with different polarities [34] and dielectric constants [35]. The results showed an obvious dependence of both reactions with OBQ and DDQ on polarity and dielectric constants of solvents. Generally, the absorbances of the reaction mixtures carried out in polar solvents, such as acetonitrile and methanol, were higher than those carried out in solvents with low polarity or dielectric constants, such as toluene and dioxane (Figure 3A). The absorbances in both OBQ and DDQ were found in good correlation with the dielectric constants of the solvents, as the correlation coefficients were 0.9288 and 0.9688, respectively (Figure 3B). The dependence of CER-OBQ reactions on polarity/dielectric constants of solvents could be explained by the fact that polar solvents with high dielectric constants can effectively solvate the polar species in the reaction and provide a suitable environment for the reaction occurrence, facilitating the formation of the reaction product. In case of reactions of DDQ involving CT, polar solvents favor complete electron transfer from CER (electron donor molecule) to DDQ (electron acceptor), while nonpolar solvents diminish this electron transfer. Methanol was selected for all subsequent investigations for both OBQ and DDQ even though acetonitrile gave higher absorbances because methanol is considered a greener solvent than acetonitrile [25,26,27]. 

A summary of the optimal reaction conditions is given in Table 1.

### 3.5. Molar Ratios and Reaction Mechanisms 

**Figure 4:**
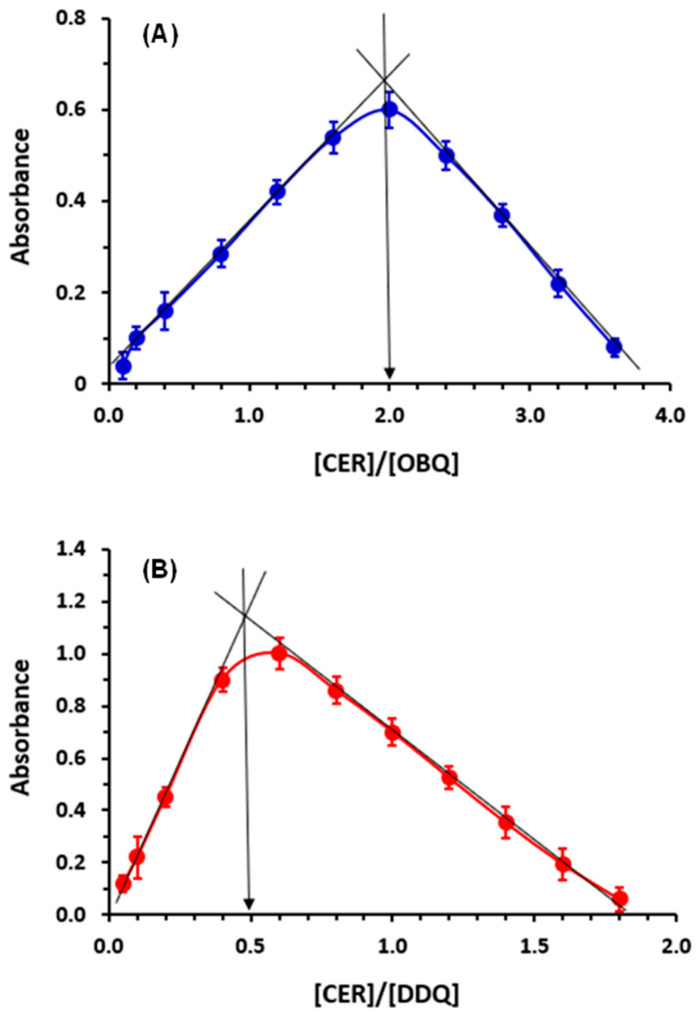
Job’s plots for reactions of CER with OBQ (**A**) and DDQ (**B**).

**Figure 5:**
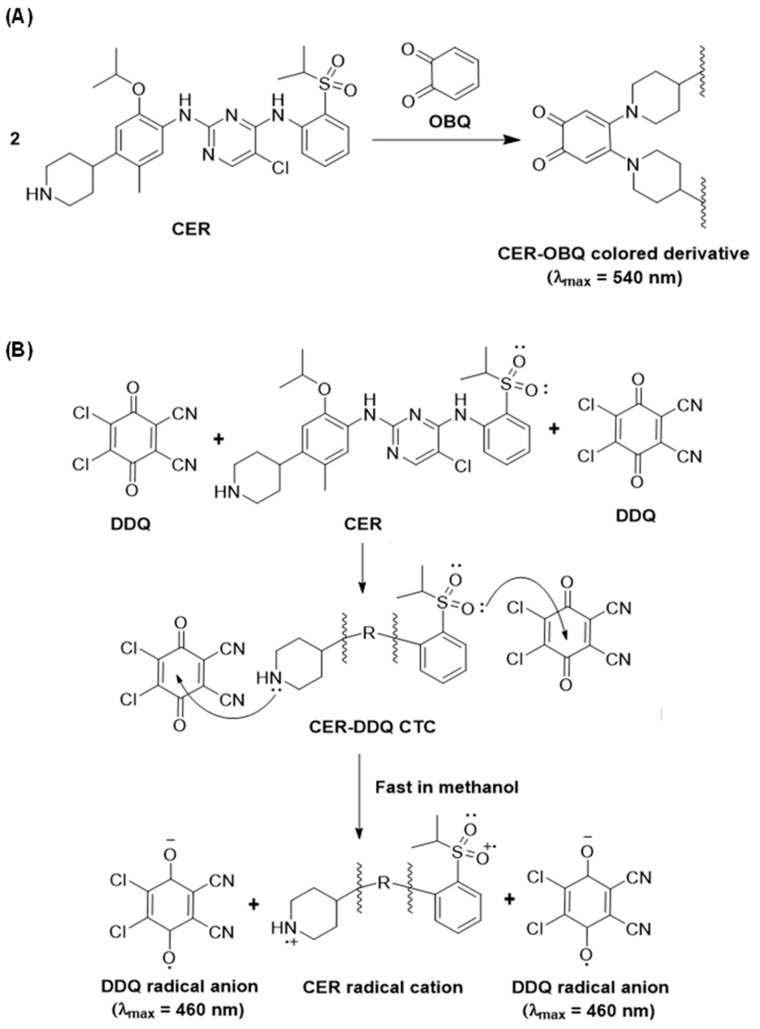
Schemes for the reaction mechanisms of CER with OBQ (**A**) and DDQ (**B**).

**Figure 6:**
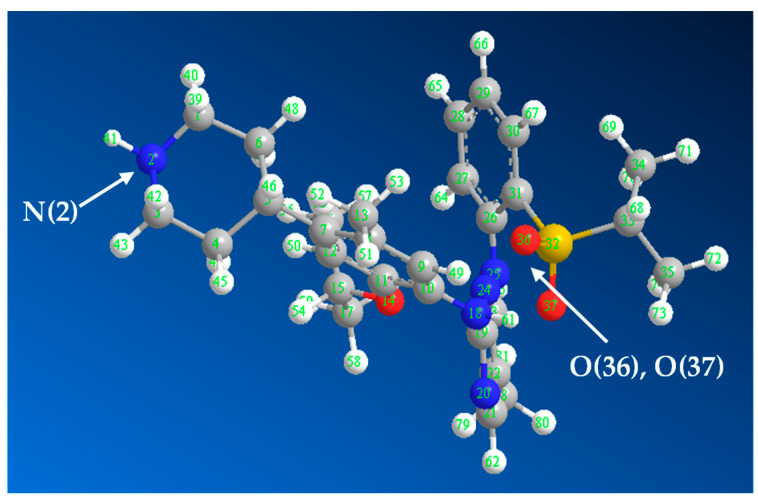
Energy-minimized CER molecule with atom numbers. arrows point to the atoms with the highest electron density, which are the piperidinyl nitrogen atom (N2) and sulfonyl oxygen atoms (O36 and O37).

The molar ratios of CER:OBQ and CER:DDQ were determined using Job’s method [17], and it was found that the ratios were 2:1 and 1:2 for reactions of CER with OBQ and DDQ, respectively (Figure 4). These ratios revealed that two molecules of CER were required for a condensation reaction with one molecule of OBQ; however, one molecule (with two electron-donating centers) of CER were required for a CT reaction with two molecules of DDQ. According to the molar ratio of CER:OBQ (2:1), the reaction mechanism was simply postulated to proceed as illustrated in Figure 5A [16,21]. For reaction with DDQ, it was necessary to identify which two electron-donating sites on the CER molecule among many possible sites contributed to the CT reaction with one molecule of DDQ. To achieve this goal, energy minimization was performed for the CER molecule, and the electron density on each atom was calculated. This was accomplished using CS Chem3D Ultra, version 16.0.0.82 (Cambridge Soft Corporation, Cambridge, MA, United States), in conjunction with molecular orbital computation tool (MOPAC) and molecular dynamics computation tool (MM2 and MMFF94). The results are presented in Figure 6 and Table 2. The highest electron density was found on nitrogen atom number 2 (N2: piperidinyl nitrogen) and oxygen atoms of the sulfonyl group (O36 and O37); both atoms had similar electron densities (Table 2). Considering the overall molar ratio and the higher electron density on the N2 atom over that on O36 and O37, accordingly, it was hypothesized that the CT reaction between one molecule of CER and two molecules of DDQ proceeded as depicted in Figure 5B [32,33].

### 3.6. Development and Validation of MW-SPMs 

Table 1 summarizes the optimal conditions for conducting the reactions of CER with OBQ and DDQ in the 96-microwell assay plate. The absorbances were measured using an absorbance microplate reader at 540 and 460 nm for reactions with OBQ and DDQ, respectively. The proposed MW-SPMs for quantitation of CER were validated according to ICH guidelines for validation of analytical procedures [36]. 

#### 3.6.1. Linear Range and Sensitivity

**Figure 7:**
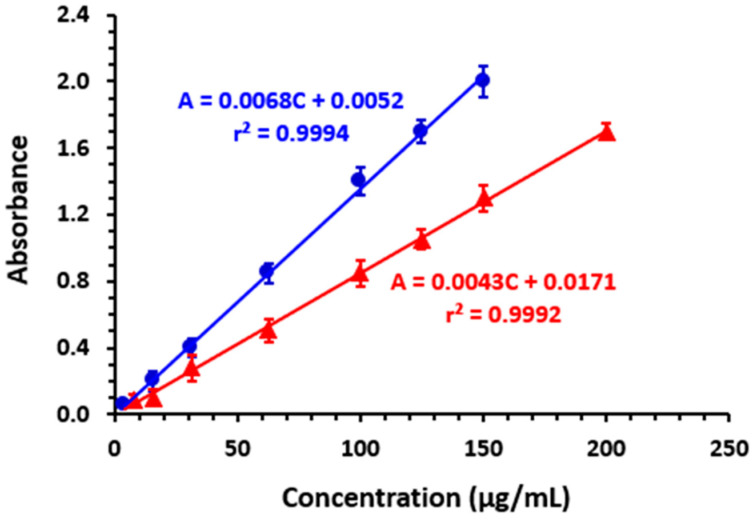
The calibration curves for quantitation of CER with the proposed MW-SPMs via its reactions with OBQ (●) and DDQ (▲). Linear regression equations and their determination coefficients (r^2^) are given on the calibration lines.

Calibration curves presenting the absorbances of CER reaction solutions with both OBQ and DDQ as a function of CER concentrations are given in Figure 7. Linear fitting of the data was performed, and linear equations along with their parameters (intercepts and slopes) and determination coefficients (r^2^) were calculated and are presented in Figure 7 and in Table 3. The proposed MW-SPMs for quantitation of CER were linear in the ranges of 5–150 and 10–200 µg/well for reactions of CER with OBQ and DDQ, respectively. The linearity of both methods was excellent, as the determination coefficients were 0.9994 and 0.9992 for methods involving reactions with OBQ and DDQ, respectively. 

The method sensitivities expressed as the limit of detection (LOD) and limit of quantitation (LOQ) were assessed using the procedures stated in the ICH guidelines for validation of analytical procedures [36]. The LOD values were 2.2 and 3.4 µg/well for methods involving reactions with OBQ and DDQ, respectively. Also, LOQ values were 6.5 and 10.2 µg/well for methods based on reactions with OBQ and DDQ, respectively.

#### 3.6.2. Precision and Accuracy

Replicate samples of CER solutions with varying levels (low, medium, and high) of concentrations (Table 4) were analyzed using both MW-SPMs, and the relative standard deviation (RSD) of the results (recovery %) was calculated and used as a measure for method precision. For intraday analysis, the replicate samples (*n* = 3) were analyzed simultaneously as a batch in one day on a single assay plate. For interday analysis, duplicate samples of each concentration level were analyzed on three consecutive days, and the means of all the results (*n* = 6), along with their RSD values, were calculated. The RSD values of both MW-SPMs did not increase over 1.6%, proving the high precision of both methods (Table 4).

The recovery (%) and error (%) of the same samples were used as measures for the methods’ accuracy. As shown in Table 4, recovery values were ≥99.6 (with errors of −0.8–2.1%) and ≥99.4 (with errors of −0.6–1.2%). These high recovery and low error values demonstrated the high accuracy of both methods for quantitation of CER.

The high precision and accuracy of the proposed MW-SPMs are likely due to several key factors. These factors are (1) the controlled environment under which the methods were conducted and the use of microscale wells for manipulation and confinement of samples, which reduced variability and enabled accurate measurements; (2) the small size of microwells, which minimized diffusion and turbulence effects, leading to more consistent and reproducible results; (3) the use of very small sample volumes possibly reducing the potential for errors or contamination, as smaller volumes are easier to handle and manipulate accurately; and (4) the methods, which involved segregating samples into individual wells, preventing cross-contamination between samples. This isolation eliminated the risk of sample mixing and ensured that measurements or reactions occurred only within the designated wells, enhancing accuracy.

#### 3.6.3. Specificity and Interference

The specificity of the proposed MW-SPMs for quantitation of CER in its Zykadia^®^ capsules without interference from the inactive ingredients contained in the capsule shell was evident because of two key reasons. The first one was conducting the measurements in the visible region of the electromagnetic light spectrum (540 and 460 nm for OBQ and DDQ, respectively), which was far away from UV-absorbing inactive ingredients. The second reason was the use of methanol for dissolution and preparation of capsule sample solutions, which dissolved only CER, leaving the inactive ingredients undissolved because they dissolve in water and do not dissolve in methanol. 

#### 3.6.4. Robustness and Ruggedness

Robustness of the proposed MW-SPMs, defined as the effect of minor changes in the methods’ variables on their analytical performance (precision and accuracy), was evaluated. These variables were OBQ and DDQ concentrations and reaction times and temperature; they were changed by 10% from the optimum values (Table 1). It was found that these changes did not negatively affect the methods’ performances; recovery values ranged from 98.5–102.3 (with RSD values of 0.8–1.4%). These results proved the suitability of the proposed methods for routine analysis of CER. 

Ruggedness was also assessed in terms of day-to-day reproducibility. The RSD values did not exceed 1.6%, proving that the proposed methods are rugged. 

### 3.7. Analysis of Zykadia^®^ Capsules and Content Uniformity Testing

Based on the satisfactory validation results mentioned above, the proposed MW-SPMs’ suitability for quantitation of CER in its commercial Zykadia^®^ capsules was evaluated. The methods were applied to determine CER concentrations in capsule samples at various predetermined levels (Table 5). The results in Table 5 display the obtained label claim percentages of mean values of 100.4 ± 1.0 and 99.6 ± 0.8% for methods involving reactions with OBQ and DDQ, respectively. These results indicate high label claim percentages, confirming the successful applicability of MW-SPMs for accurate and precise determination of CER content of Zykadia^®^ capsules.

For the content uniformity testing, USP guidelines [18] were followed. Ten capsules were individually analyzed using both the OBQ and DDQ methods for their CER contents. The acceptance values were calculated and found to be 3.6 and 3.1, respectively (Table 6). These values were less than the maximum allowed average value (15), confirming the excellent uniformity of CER contents in capsules

### 3.8. Greenness Levels of MW-SPMs

Primarily, MW-SPMs assisted with microplate readers adhere to the principles of green analytical chemistry (GAC) by miniaturizing analytical processes. This approach offers several advantages over the traditional spectrophotometric analytical approach using volumetric flasks and cuvettes, including the use of lower sample and reagent volumes, as well as reduced waste production. To evaluate the environmental sustainability of the proposed MW-SPMs, three effective metric tools were employed: the Analytical Eco-Scale (AES) [37], the Green Analytical Procedure Index (GAPI) [38], and AGREE-analytical greenness metric approach and software, version v. 0.5 beta 2020 [39]. These tools provide a precise and comprehensive assessment of the greenness of analytical procedures, and their evaluation parameters and results can be found in their corresponding published articles [37,38,39].

The results obtained from the AES are shown in Table 7 for both the OBQ- and DDQ-based methods, as they involved the same reagent category, procedures, and operating conditions. The penalty points (PPs) due to amount of solvent (methanol) and reagents (OBQ and DDQ) were both, with a subtotal of 2 PPs. The PP subtotal due to the hazardous effect of methanol and reagents for each method was 6 PPs. The parameters of energy consumption by instruments and occupational hazards did not gain any PPs because they met the GAC guidelines. The parameters of waste production and treatment were given sub-totals of 1 and 3 PPs, respectively. This score was assigned because the proposed MW-SPMs produced <1 mL of waste per sample, and the waste was not treated. The total PPs for each MW-SPM was 12, and accordingly, the assays’ eco-scale scores were 88 (100–12). This high score revealed the excellent level of ecofriendship of the proposed MW-SPMs, as per the metric guidelines [37].

**Figure 8:**
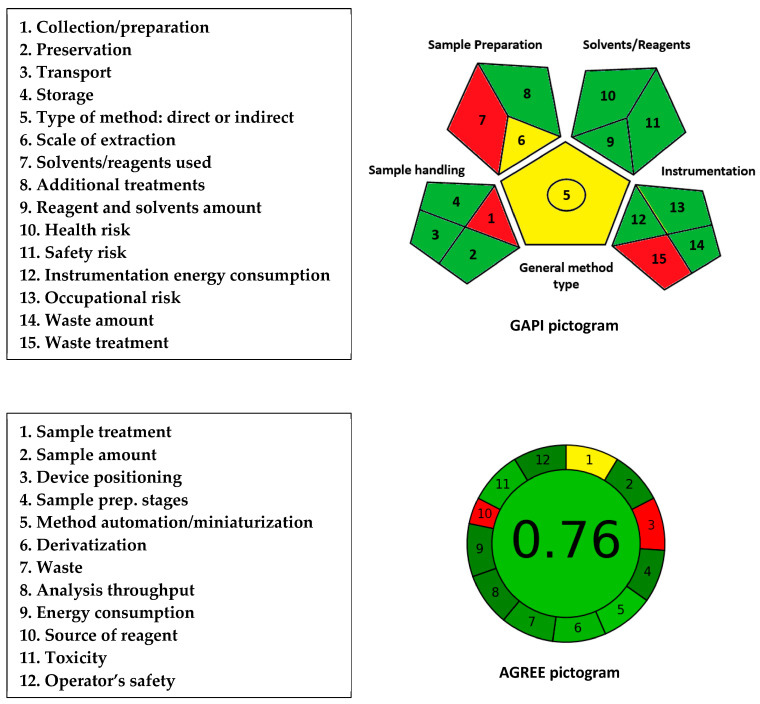
Pictograms of GAPI and AGREE analyses for evaluation of the greenness of the proposed SPMs for quantitation of CER.

The results obtained using the GAPI tool regarding 15 parameters (pictogram) are shown in Figure 8. Among the 15 parameters, 3 parameters (1, 7, and 15) were given red colors in the pictogram. These parameters were assigned a red color because sample collection/preparation was carried out in an offline manner, methanol was used for sample preparation, and the assays’ waste was not treated, respectively. Parameters 5 and 6 were assigned yellow because the methods were applicable to quantitative analysis and the extraction of samples was performed in microscale, respectively. The other parameters were given a green color because they fulfilled the requirements of green procedures, as per the tool guidelines [38].

The pictogram obtained with AGREE is shown in Figure 8. Parameter 1 (corresponding to sample treatment) was assigned a yellow color because the sample treatment was manually carried out. Parameter 3 (corresponding to device positioning either online or offline) and parameter 10 (corresponding to source of reagent) took a red color because the analysis was conducted with a plate reader in an offline way and because of the use of DDQ as a chemical source of reagent, respectively. The other parameters were assigned a green color, and the total score was 0.76 out of 1. This score confirmed the high greenness level of the proposed MW-SPMs.

In conclusion, the results of the three tools confirmed the greenness of the proposed methods and their adherence to GAC principles. 

### 3.9. Comparison of Greenness Levels of MW-SPMs with Reported Methods

**Figure 9:**
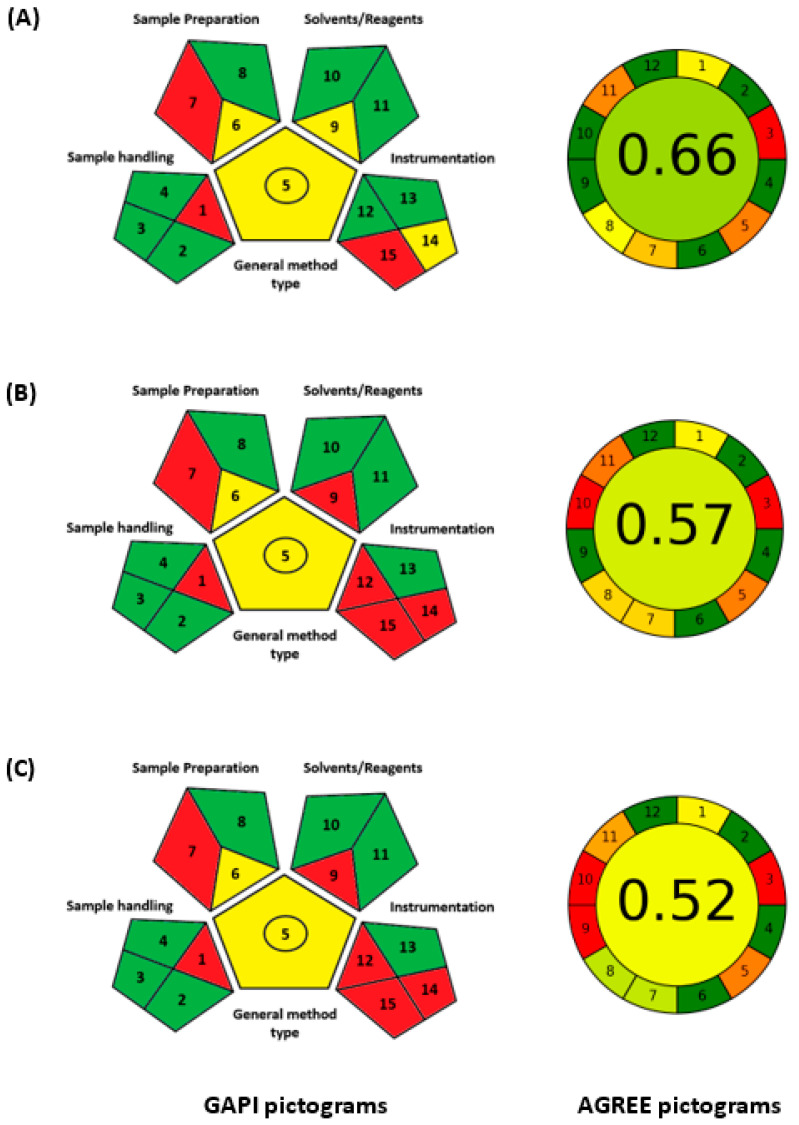
Comparative results of GAPI (left hand side) and AGREE (right hand side) metric tools for evaluation of the greenness of the reported methods for CER. The parameters’ numbers and definitions of each parameter of both GAPI and AGREE are given in Figure 8. Panels (**A**), (**B**), and (**C**) refer to references [9], [10], and [11], respectively.

The greenness levels of the proposed MW-SPMs were compared with those of the reported liquid chromatographic methods for CER [9,10,11]. The comparison was conducted by using the GAPI and AGREE tools, and the pictograms obtained for the reported methods using these tools are presented in Figure 9. The GAPI tool gave similar greenness levels for the reported methods, and these levels were lower than those of the proposed MW-SPMs, particularly for parameters 9, 12, and 14 (corresponding to reagent and solvent amount, instrument energy consumption, and waste amount, respectively). This observation was interpretable based on the fact that liquid chromatography consumes larger amounts of solvents and reagents, uses more energy for operation, and produces larger volumes of waste than the proposed MW-SPMs. 

The AGREE tool, the most recent and comprehensive tool, revealed that the proposed MW-SPMs had the highest level of greenness (0.76) compared with 0.66, 0.57, and 0.52 for the reported methods [9,10,11], respectively. The LC-MS/MS method [11] gave the lowest greenness level because it had the highest energy consumption among the other HPLC-UV methods [9,10]. The superior greenness level of the proposed MW-SPMs over the reported methods was mainly due to the volume of samples and, consequently, the volume of waste produced in the proposed methods compared with the reported ones. It is wise to mention that the waste outcome of the proposed method was 200 µL/sample compared with 4–8 mL/sample for the reported liquid chromatographic methods. In addition, the reported HPLC-UV and LC-MS/MS methods used larger volumes of solvents (methanol, and acetonitrile), formic acid, and different buffers in the mobile phase, which negatively reflected on their greenness levels, compared with the proposed MW-SPMs, which used smaller volumes of samples and reagents (total 200 µL/sample). 

### 3.10. Throughputs of MW-SPMs

In principle, microwell methods have gained significant attention in pharmaceutical analysis because of their ability to simultaneously analyze and manipulate large numbers of individual entities at high throughput. These methods involve the use of arrays of microscale wells or compartments, where each well can accommodate a single entity, such as a molecule target analyte [13,14,15]. The throughput of the proposed MW-SPMs was assessed considering the use of 96-well plates and reaction times of 5 and 10 min in cases of using OBQ and DDQ, respectively. It was possible for an analyst to simultaneously and comfortably process at least five plates as a batch. In these circumstances, 1920 samples could be processed per hour (5 plates × 96 wells × 4 rounds/h). This high throughput could be further improved via different approaches. These approaches include using plates with a greater number of wells (384, 1536, or 3456 wells) and automation of the process using robots. 

## 4. Conclusions

This study described the development and validation of two MW-SPMs for the quantitation of CER in its capsules. These two methods involved the reaction of CER with two different benzoquinolone reagents (OBQ and DDQ) via two different reaction mechanisms. These reactions were condensation and CT reactions for OBQ and DDQ, respectively. The reactions were carried out in 96-well transparent plates, and the absorbances of the colored reaction solutions were measured with an absorbance microplate reader. Both methods were developed for the first time for CER, and they involved very simple procedures and fulfilled the requirements of GAC practices in quality control laboratories in pharmaceutical industries in terms of eco-friendliness and health safety. Both assays were applied successfully to the determination of CER content in capsules and drug uniformity testing. In addition, both methods had high analytical throughput, as they could enable an analyst to analyze ~1920 samples per hour with convenience and comfort. Furthermore, the methods had high sensitivity for quantitation of low concentrations of CER with acceptable accuracy and precision. The overall results of the present work broaden the perspective of the efficient employment of microwell assays assisted with plate readers for quantitation of drugs and prove their convenient applications in the pharmaceutical industry.

## Figures and Tables

**Table 1:** Optimization of experimental conditions of MW-SPMs for CER via its reactions with OBQ and DDQ.

	OBQ		DDQ	
Condition	Studied Range	Optimum Value	Studied Range	Optimum Value
Reagent conc. (%, *w*/*v*)	0.05–1.2	0.5	0.05–1.2	1
Solvent	Different ^a^	Methanol	Different ^a^	Methanol
Reaction time (min)	0–25	10	0–25	5
λ_max_ (nm)	400–800	540	400–800	460

^a^ Solvents used were acetonitrile, methanol, ethanol, propanol, dichloromethane, dichloroethane, toluene, and dioxane.

**Table 2:** Atom types, numbers, and calculated charges on energy-minimized CER.

Atom Number	Atom Type	Charge	Atom Number	Atom Type	Charge
C(1)	Alkyl carbon, SP3	0.27	N(24)	Aromatic nitrogen with s lone pair	−0.62
N(2)	Amine nitrogen	−0.9	N(25)	Enamine or aniline nitrogen, Deloc. LP	−0.6
C(3)	Alkyl carbon, SP3	0.27	C(26)	Aromatic carbon, in benzene, pyridine	0.1
C(4)	Alkyl carbon, SP3	0	C(27)–C(30)	Aromatic carbon, in benzene, pyridine	−0.15
C(5)	Alkyl carbon, SP3	0.1435	C(31)	Aromatic carbon, in benzene, pyridine	−0.009
C(6)	Alkyl carbon, SP3	0	S(32)	Sulfone sulfur	1.2038
C(7)–C8	Aromatic carbon, in benzene, pyridine	−0.1435	C(33)	Alkyl carbon, SP3	0.1052
C(9)	Aromatic carbon, in benzene, pyridine	−0.15	C(34)–C(35)	Alkyl carbon, SP3	0
C(10)	Aromatic carbon, in benzene, pyridine	0.1	O(36)–O(37)	Terminal O’s on sulfur	−0.65
C(11)	Aromatic carbon, in benzene, pyridine	0.0825	C(38)	Alkyl carbon, SP3	0.36
C(12)	Aromatic carbon, in benzene, pyridine	−0.15	H(39)–H(40)	H attached to C	0
C(13)	Alkyl carbon, SP3	0.1435	H(41)	Generic H on SP3 nitrogen in amine	0.36
O(14)	Ether oxygen	−0.3625	H(42)–H(48)	H attached to C	0
C(15)	Alkyl carbon, SP3	0.28	H(49)–H(50)	H on enamine N	0.15
C(16)–C(17)	Alkyl carbon, SP3	0	H(51)–H(60)	H attached to C	0
N(18)	Enamine or aniline nitrogen, Deloc. LP	−0.6	H(61)	Hydrogen in H-N-C=N moiety	0.4
C(19)	Aromatic carbon, in benzene, pyridine	0.72	H(62)	H attached to C	0.15
N(20)	Aromatic carbon, in benzene, pyridine	−0.62	H(63)	Hydrogen on enamine nitrogen	0.4
C(21)	Aromatic carbon, in benzene, pyridine	0.16	H(64)–H(67)	H attached to C	0.15
C(22)	Aromatic carbon, in benzene, pyridine	−0.1435	H(68)–H(81)	H attached to C	0
C(23)	Aromatic carbon, in benzene, pyridine	0.41			

**Table 3:** Regression and statistical parameters for the determination of CER with the proposed MW-SPMs via reactions with OBQ and DDQ.

Parameter	Value	
	OBQ	DDQ
Linear range (µg/well)	5–150	10–200
Intercept	0.0052	0.0171
Slope	0.0068	0.0043
Determination coefficient (r^2^)	0.9994	0.9992
Limit of detection (µg/well)	2.2	3.4
Limit of quantitation (µg/well)	6.5	10.2

**Table 4:** Precision and accuracy of MW-SPMs for the quantitation of CER via its reactions with OBQ and DDQ.

CER Concentration (µg/well)	Intraday (*n* = 3)		Interday (*n* = 6)	
	Recovery (% ± RSD)	Error (%)	Recovery (% ± RSD)	Error (%)
OBQ				
20	101.2 ± 1.3	1.2	99.6 ± 1.3	−0.4
80	99.8 ± 1.2	−0.4	100.6 ± 1.1	0.6
120	100.1 ± 1.4	0.1	99.8 ± 1.3	−0.2
DDQ				
25	102.1 ± 1.2	2.1	101.2 ± 1.2	1.2
100	99.4 ± 0.9	−0.8	99.5 ± 0.8	−0.6
180	100.4 ± 1.3	0.4	100.8 ± 1.6	0.8

**Table 5:** Application of MW-SPMs for the quantitation of CER in Zykadia^®^ capsules via its reactions with OBQ and DDQ.

Nominated CER Concentration (µg/well)		Label Claim (% ± RSD) ^a^	
		OBQ	DDQ
50		99.6 ± 1.3	100.4 ± 1.4
100		100.2 ± 1.4	98.8 ± 1.2
150		101.5 ± 1.6	99.5 ± 1.0
	Mean	100.4 ± 1.0	99.6 ± 0.8

^a^ Average of 3 determinations.

**Table 6:** Content uniformity testing of Zykadia^®^ capsules using the proposed MW-SPMs via CER reactions with OBQ and DDQ.

Capsule Number	Label Claim (%)	
	OBQ	DDQ
1	99.5 ± 1.2	101.5 ± 1.5
2	99.2 ± 2.3	100.2 ± 1.2
3	102.5 ± 1.8	98.8 ± 0.8
4	98.8 ± 1.4	100.2 ± 1.4
5	97.9 ± 1.9	99.5 ± 2.1
6	100.6 ± 1.5	97.8 ± 2.2
7	101.2 ± 0.9	101.7 ± 1.4
8	98.4 ± 1.3	100.2 ± 0.6
9	100.8 ± 0.9	99.5 ± 0.8
10	98.2 ± 1.3	101.8 ± 1.2
Mean	99.7	100.1
SD	1.5	1.3
Acceptance value	3.6	3.1
Maximum allowed value	15	15

**Table 7:** Analytical Eco-Scale for assessing the greenness of the proposed MW-SPMs for the quantitation of CER via its reactions with OBQ and DDQ.

Eco-Scale Score Parameters	Penalty Points (PPs)
Amount of solvent/reagent	
Solvent: <1 mL (mL (g) per sample)	1
Reagent: <1 mL (mL (g) per sample)	1
	∑ = 2
Hazard of solvent/reagent	
Solvent: methanol	3
Reagent: OBQ and DDQ	3
	∑ = 6
Instrument: energy used (kWh per sample)	
Microplate reader	0
	∑ = 0
Occupational hazards	
Analytical process is hermetic	0
Emission of vapors and gases to the air	0
	∑ = 0
Waste	
Production (<1 mL (g) per sample)	1
Treatment (no treatment involved)	3
	∑ = 4
Total PPs	12
Eco-Scale score	88

## Data Availability

All data are available from the corresponding author (idarwish@ksu.edu.sa).

## References

[1] American Cancer Society Key Statistics for Lung Cancer. https://www.cancer.org/cancer/types/lung-cancer/about/key-statistics.html#:~:text=Overall%2C%20the%20chance%20that%20a,t%2C%20the%20risk%20is%20lower.

[2] Anne C., Chiang R.S.H. (2021). Lung Cancer: New Understandings and Therapies.

[3] Burotto M., Manasanch E.E., Wilkerson J., Fojo T. (2015). Gefitinib and erlotinib in metastatic non-small cell lung cancer: A meta-analysis of toxicity and efficacy of randomized clinical trials. Oncologist.

[4] Yang Z., Hackshaw A., Feng Q., Fu X., Zhang Y., Mao C., Tang J. (2017). Comparison of gefitinib, erlotinib and afatinib in non-small cell lung cancer: A meta-analysis. Int. J. Cancer.

[5] Lei Y., Lei Y., Shi X., Wang J. (2022). EML4-ALK fusion gene in non-small cell lung cancer (Review). Oncol. Lett..

[6] Shaw A.T., Kim D.W., Mehra R., Tan D.S., Felip E., Chow L.Q., Camidge D.R., Vansteenkiste J., Sharma S., De Pas T. (2014). Ceritinib in ALK-rearranged non-small-cell lung cancer. N. Engl. J. Med..

[7] U.S. FDA. Food and Drug Administration FDA Broadens Ceritinib Indication to Previously Untreated ALK-Positive Metastatic NSCLC. https://www.fda.gov/drugs/resources-information-approved-drugs/fda-broadens-ceritinib-indication-previously-untreated-alk-positive-metastatic-nsclc#:~:text=On%20May%2026%2C%202017%2C%20the,by%20an%20FDA%2Dapproved%20test.

[8] Tian W., Zhang P., Yuan Y., Deng X.-H., Yue R., Ge X.-Z. (2020). Efficacy and safety of ceritinib in anaplastic lymphoma kinase-rearranged non-small cell lung cancer: A systematic review and meta-analysis. J. Clin. Pharm. Ther..

[9] Kumar C.N., Prathyusha V., Kannappan N. (2014). A novel validated stability indicating RP-HPLC method development for the estimation of ceritinib in its bulk and finished dosage form as per ICH guidelines. Pharm. Lett..

[10] Adhao V.S., Sharma J., Thakre M. (2017). Development and validation of stability indicating RP-HPLC method for determination of ceritinib. Indones. J. Pharm..

[11] Antolčić M., Runjea M., Galić N. (2020). A simple and sensitive LC-MS/MS method for determination and quantification of potential genotoxic impurities in the ceritinib active pharmaceutical ingredient. Anal. Methods.

[12] Liu G., Lin J.-M. (2016). Microplate-based assays: The future of pharmaceutical analysis. Trends Anal. Chem..

[13] Singh P., Singh B. (2017). Microwell spectrophotometry: A green analytical technique for pharmaceutical analysis. J. Pharm. Anal..

[14] Welch C.J. (2019). High throughput analysis enables high throughput experimentation in pharmaceutical process research. React. Chem. Eng..

[15] Roschangar F., Sheldonb R.A., Senanayakea C.H. (2015). Overcoming barriers to green chemistry in the pharmaceutical industry—The Green Aspiration Level™ concept. Green Chem..

[16] Alshehri M.M., Darwish I.A., Kassem M.G., Maher H.M., Alzoman N.Z. (2014). Development of novel microwell-plate spectrophotometric assay for determination of varenicline via its reaction with cyclohexa-3,5-diene-1,2-dione. Dig. J. Nanomater. Biostructures.

[17] Job P. (1964). Advanced Physicochemical Experiments.

[18] Rockville M. (2007). The United States Pharmacopoeia 30, the National Formulary 25 US Pharmacopeial Convention. Electron. Version.

[19] Görög S. (2018). Ultraviolet-Visible Spectrophotometry in Pharmaceutical Analysis.

[20] Upadhyay K., Tamrakar R.K., Dubey V. (2013). Development of Spectrophotometric Methods for Pharmaceutical Analysis.

[21] Askal H.F., Refaat I.H., Darwish I.A., Marzouq M.A. (2011). Spectrophotometric determination of lomefloxacin in its pharmaceutical dosage forms. Asian J. Chem..

[22] Passos M.L.C., Sarraguça M.C., Saraiva L.M.F.S., Rao T.P., Biju V.M., Worsfold P., Poole C., Townshend A., Miró M. (2019). Spectrophotometry|Organic Compounds. Encyclopedia of Analytical Science.

[23] Alsharif M.A., Raja Q.A., Abdul Majeed N., Jassas R.S., Alsimaree A.A., Sadiq A., Naeem N., Mughal E.U., Alsantali R.I., Moussa Z. (2021). DDQ as a versatile and easily recyclable oxidant: A systematic review. RSC Adv..

[24] Berto S., Chiavazza E., Ribotta V., Daniele P.G., Barolo C., Giacomino A., Vione D., Malandrino M. (2015). Charge-transfer complexes of 2,3-dichloro-5,6-dicyano-1,4-benzoquinone with amino molecules in polar solvents. Spectrochim. Acta Part A Mol. Biomol. Spectrosc..

[25] Dunn P.J., Wells A.S., Williams M.T. (2010). Green Chemistry in the Pharmaceutical Industry.

[26] Agbenyega J. Green Chemistry in the Pharma Industry: Sustainable Pastures for Those Who Innovate. https://www.cas.org/resources/cas-insights/sustainability/green-chemistry-pharma-industry.

[27] Msingh R., Pramanik R., Hazra S. (2021). Role of green chemistry in pharmaceutical industry: A review. J. Univ. Shanghai Sci. Technol..

[28] Wang P.G. (2019). High-Throughput Analysis in the Pharmaceutical Industry.

[29] Mennen S.M., Alhambra C., Allen C.L., Barberis M., Berritt S., Brandt T.A., Campbell A.D., Castañón J., Cherney A.H., Christensen M. (2019). The evolution of high-throughput experimentation in pharmaceutical development and perspectives on the future. Org. Process Res. Dev..

[30] Janzen W.P. (2016). High Throughput Screening Methods and Protocols.

[31] Darwish I.A., Alzoman N.Z. (2023). Development and validation of green and high-throughput microwell spectrophotometric assay for the determination of selective serotonin reuptake inhibitors in their pharmaceutical dosage forms. Molecules.

[32] Khalil N.Y., Al Qhatani M.N., Al Qubaisi K.A., Alqarni M., Sayed A.Y., Darwish I.A. (2022). Development of two innovative 96-microwell-based spectrophotometric assays with high throughput for determination of fluoroquinolone antibiotics in their pharmaceutical formulations. Lat. Am. J. Pharm..

[33] Darwish I.A., Khalil N.Y., Alsaif N.A., Herqash R.N., Sayed A.Y.A., Abdel-Rahman H.M. (2021). Charge-transfer complex of linifanib with 2,3-dichloro-3,5-dicyano-1,4-benzoquinone: Synthesis, spectroscopic characterization, computational molecular modelling and application in the development of novel 96-microwell spectrophotometric assay. Drug Des. Devel. Ther..

[34] Polarity Index. http://macro.lsu.edu/howto/solvents/polarity%20index.htm.

[35] University of Washington Dielectric Constant of Common Solvents. https://depts.washington.edu/eooptic/linkfiles/dielectric_chart%5B1%5D.pdf.

[36] ICH (2022). International Council for Harmonisation of Technical Requirements for Pharmaceuticals for Human Use, ICH Harmonised Guideline, Validation of Analytical Procedure: Q2(R2).

[37] Gałuszka A., Konieczka P., Migaszewski Z.M., Namies’nik J. (2012). Analytical eco-scale for assessing the greenness of analytical procedures. Trends Anal. Chem..

[38] Płotka-Wasylka J. (2018). A new tool for the evaluation of the analytical procedure: Green Analytical Procedure Index. Talanta.

[39] Pena-Pereira F., Wojnowski W., Tobiszewski M. (2020). AGREE-analytical greenness metric approach and software. Anal. Chem..

